# Luminescence Lifetime-Based Sensing of Water Turbidity

**DOI:** 10.1021/acssensors.5c00849

**Published:** 2025-05-07

**Authors:** Ya Jie Knöbl, Iman Nakhli, María del Mar Darder, Guillermo Orellana

**Affiliations:** Chemical Optosensors & Applied Photochemistry Group (GSOLFA), Dpmt. of Organic Chemistry, Faculty of Chemistry, 16734 Complutense University of Madrid, Madrid 28040, Spain

**Keywords:** lifetime-based sensor, water quality monitoring, turbidity sensor, phase-sensitive detection, dual lifetime referencing

## Abstract

Current commercial turbidity sensors may be costly, bulky, or fragile. As such, many research groups have investigated alternative methods for the accurate determination of this essential water quality parameter. This work describes a new sensor based on luminescence measurements under a dual-lifetime reference scheme. By letting the turbid water pass between two dye layers with similar absorption and emission features but widely different emission lifetimes, an overall luminescence phase shift is measured, the magnitude of which depends on the turbidity. Dimethyl 2,5-bis­(cyclohexylamino)­terephthalate (BCT) is the reference fluorophore placed on the optical fiber tip after immobilization in a thin poly­(vinyl chloride) (PVC) layer. The indicator luminophore, tris­(4,7-diphenyl-1,10-phenanthroline)­ruthenium­(II) (RD3), embedded into poly­(ethyl 2-cyanoacrylate) (PCA), is separated from the reference layer by a user-selectable distance (1–2 cm). With increasing turbidity, the emission intensity of the indicator dye layer decreases, while the fluorescence intensity of the reference layer remains constant. In this way, the ratio between the two emission intensities is translated into changes in the lifetime (and phase shift) of the composite emission. The sensor’s working range depends on the distance between the two dye layers. The sensor is capable of detecting turbidity levels in the range of 0–1000 NTU, 0–500 NTU, and 0–300 NTU for 1, 1.5, and 2 cm optical pathlengths, respectively, with an accuracy of 1 NTU (0.3 NTU between 0 and 10 NTU), limited by the accuracy of the turbidity standards. Shorter pathlengths allow the measurement of higher turbidity. The temperature-dependent response is instantaneous and devoid of dissolved O_2_ and chlorophyll interferences. The sensor has been tested in a real-world environment for 11 days with good performance.

## Graphical abstract

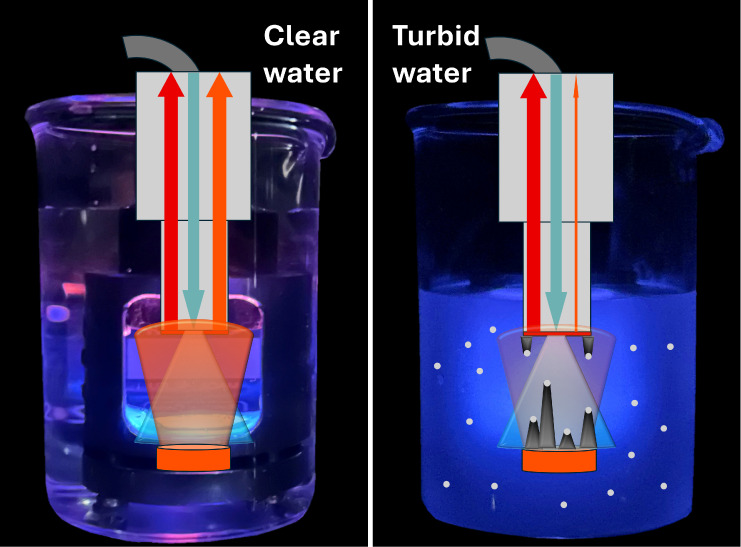

Turbidity, among other important water quality parameters, is commonly monitored in natural water bodies. It refers to the effect of suspended particles, organic or inorganic, microorganisms, and other solid matter blocking sunlight and lowering the transparency of water.^1^ This obstruction raises the water temperature, lowers the photosynthetic rate, and leads to a decrease in the dissolved O_2_ level and water stratification.^2,3^ The increase in turbidity in an aqueous ecosystem can be attributed to many factors, spanning from those of natural origin, like erosion, to those from human sources, like discharges from wastewater treatments or agricultural runoff.^4^

Visual assessment methods, such as the Secchi disk, have been used to estimate water turbidity.^5^ However, to avoid subjective interpretation by the operator, methods based on turbidimetry or nephelometry are nowadays standard.^6^ Both techniques rely on a beam of light passing through a water sample to measure its attenuation or reflectance. The difference between them lies in the detector placement.^7^ In turbidimetry, the detector is aligned with the incident beam of light (θ = 0°), the attenuation of which is measured. In nephelometry, the scattered light is measured at an angle to the incident beam and is further divided into three categories: forward scattering (0° ≤ θ < 90°), side scattering (θ = 90°), and backscattering (90° < θ ≤ 180°).^7^ Only turbidimetry and side scattering nephelometry are the standard methods sanctioned by the ISO 7027 norm, together with defined light sources and wavelengths.

Commercial turbidity sensors operating under the aforementioned techniques, as well as those based on acoustic backscattering, are often costly, bulky, and fragile.^8–10^ Therefore, different sensor setups have been developed to address these issues. Such devices involve fiber Bragg gratings or surface plasmon resonance on optical fibers as the sensing elements,^11^ multiple light sources and detectors,^12–15^ nonstandard detector types,^16–18^ or the use of satellite data and machine learning^19–21^ to determine turbidity. While some of these solutions are sophisticated on their own, their underlying principles often make them less amenable to share their electronics with those of other sensors for key water quality parameters, such as dissolved O_2_, pH, or conductivity. To capitalize on the latest technology for robust dissolved O_2_ measurements using luminescence lifetime-based sensing in the frequency domain, we set out to design and realize an optical turbidity sensor based on this technique.

The luminescence lifetime (τ) and the luminescence phase shift (ϕ) are related by eq 1,

1 tan(ϕ)=2πfτ

where *f* is the modulation frequency of the sinusoidal excitation light. The novel sensor consists of two blue-absorbing, red-emitting luminescent dyes placed at a distance from each other. Using the so-called “dual lifetime referencing” (DLR) scheme,^23,24^ the long-lived emission of one of the two dyes is perturbed by the short-lived fluorophore to a degree that is dependent on the water turbidity. The resulting compounded luminescence can be measured by the very same optoelectronic units employed for dissolved O_2_ monitoring.^25^

## Experimental Section

Syntheses of the luminophores, reactants, solvents, instrumentation, experimental procedures for the spectroscopic measurements, and a scheme of the turbidity measurement setup (Figure S1) are described in the Supporting Information.

### Fabrication of the Luminescent Monolith

In a 5 mL vial, 1.20 mg of tris­(4,7-diphenyl-1,10-phenanthroline)­ruthenium­(II) bis­(hexafluorophosphate) (RD3, Chart [Fig chart1]) is dissolved in 50 μL of acetonitrile. Then, 1.00 mL of ethyl 2-cyanoacrylate (Henkel Loctite Super Glue-3, Düsseldorf, Germany) is added, and the mixture is stirred without delay for 5 s with the pipet tip. After that, two aliquots of 450 μL of the mixture are placed into each of two 1 cm i.d. HPLC vials and evacuated to 0.1 Torr overnight. The vials are then placed in a closed container under a water vapor-saturated atmosphere and allowed to cure for 7 days. Following that, the vials are carefully broken to remove the resulting polymer monolith. With the aid of an Allied MultiPrep System-8 polisher (Cerritos, CA), the monolith is reduced to a thickness of 2.00 mm with a final polishing using sandpaper with 1 μm diamond grain.

**1 chart1:**
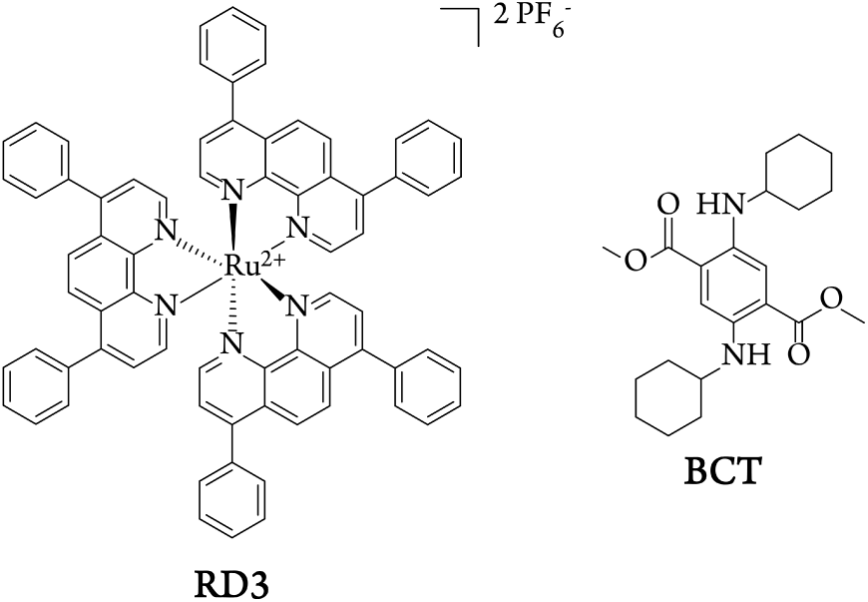
Structures of the Luminescent Ruthenium­(II) Complex RD3 and the Fluorescent Organic Dye BCT

### Fabrication of the Fluorescent Membrane

First, a 10% (w/v) poly­(vinyl chloride) (PVC) solution is prepared by weighing 5.00 g of PVC powder (Selectophore, Merck, Darmstadt, Germany), adding 50 mL of tetrahydrofuran (THF), and heating the mixture at 60 °C for 3 h under vigorous magnetic stirring. In the meantime, a stock solution of dimethyl 2,5-bis­(cyclohexylamino)­terephthalate (BCT, Chart [Fig chart1]) is prepared by dissolving 2.00 mg of BCT in 1.00 mL of THF and stirring the mixture for 30 min. A cocktail of 5.7 μL of the BCT stock solution and 1.99 mL of the 10% PVC solution is prepared for a final BCT concentration of 15 μmol L^–1^. This mixture is stirred for 1 h in the dark before being transferred to a 28 mm i.d. glass Petri dish (“40 mm” Steriplan, Duran, Wertheim/Main, Germany) while avoiding bubble formation. The Petri dish is placed into a closed container with a saturated THF atmosphere for 24 h to allow film formation (147 ± 6 μm, *n* = 5). The film is carefully removed from the Petri dish and placed into a zip bag until usage.

### Fabrication and Assembly of the Sensitive Terminal

The sensitive terminal heads (Figure 1) with different heights to produce the target optical path lengths are 3D-printed on a BCN3D (Barcelona, Spain) Sigma D25 printer using PET-G filament, while the housing is home manufactured in 316 stainless steel (UCM Central Support Research Facilities). The detachable bottom part of the housing is polished to mirror finishing.

**Figure 1:**
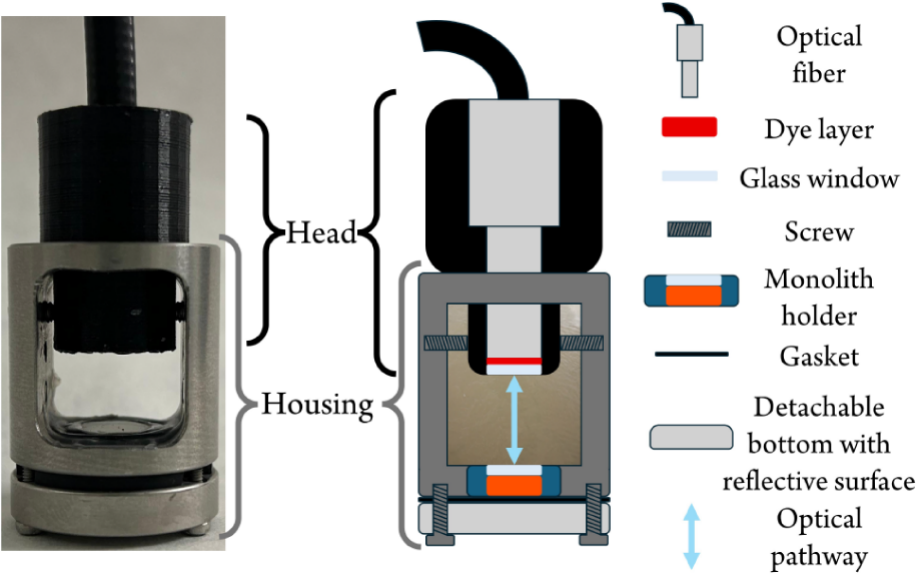
Sensitive terminal (left) and schematic depiction (right) with its components that are placed into the turbid water sample.

A 9.0 mm disk is cut from the fluorescent BCT film and installed into the sensitive terminal head on top of a 10 mm glass window (Edmund Optics, Barrington, NJ). The glass-plastic junction is sealed with neutral silicone (DOWSIL 3140, Dow, Midland, MI). The luminescent monolith is placed into a 3D-printed holder under another 10 mm glass window (Figure 1). The glass-plastic junction is also sealed with the same silicone. The monolith holder assembly is placed at the bottom of the stainless steel housing and sealed with silicone before placing a 24 × 14 × 2 mm rubber gasket and fixing the detachable bottom part of the housing with four screws.

After the common end of the optical fiber bundle is introduced (see Supporting Information) into the sensitive terminal head, the latter is inserted into the housing and secured with two horizontal screws (Figure 1).

## Results and Discussion

### Background

Being that turbidity (*T*) is a physical parameter, transduction involving luminescent indicator dyes is not straightforward. Attenuation of the excitation light passing through a turbid sample would lead to a decrease of the emission intensity of a luminescent material. However, the indicator emission lifetime (and therefore the phase shift) will not change. To convert luminescence intensity changes into phase shift variations, the so-called “dual lifetime referencing” (DLR) method is employed (Figure 2).^23,24^ According to DLR, the long lifetime (large phase shift) of a luminophore can be perturbed by the short-lived emission (zero phase shift) of a fluorophore as long as the two dyes absorb in overlapping spectral regions and their emissions are collected simultaneously. When this condition is met, the measured lifetime is dependent on the ratio of the emission intensities of the two luminophores (eq 2),^24^

2 $$\cot\left(\phi \right)=\cot\left(\phi_{L}\right)+\frac{1}{\sin\left(\phi_{L}\right)}\frac{A_{S}}{A_{L}}$$

where ϕ is the measured phase shift of the compound emission, ϕ_L_ is the phase shift of the long-τ luminescent dye, and *A*_S_ and *A*_L_ are the amplitudes of the sinusoidally modulated emissions of the short-τ and long-τ dye, respectively. Eq 2 assumes that ϕ_L_ is kept constant and that the frequency (*f*) of the sinusoidally modulated excitation (blue) light is optimized for the long-τ dye, so that tan­(ϕ_L_) equals 1 and the phase shift of the short-τ luminescent dye (ϕ_S_) is 0.^24^ This assumption holds whenever the difference in the emission lifetimes is larger than 2 orders of magnitude. Changes in the ratio of the emission intensities lead to variations in the analytical signal (ϕ) and therefore of the measured luminescence lifetime.

**Figure 2:**
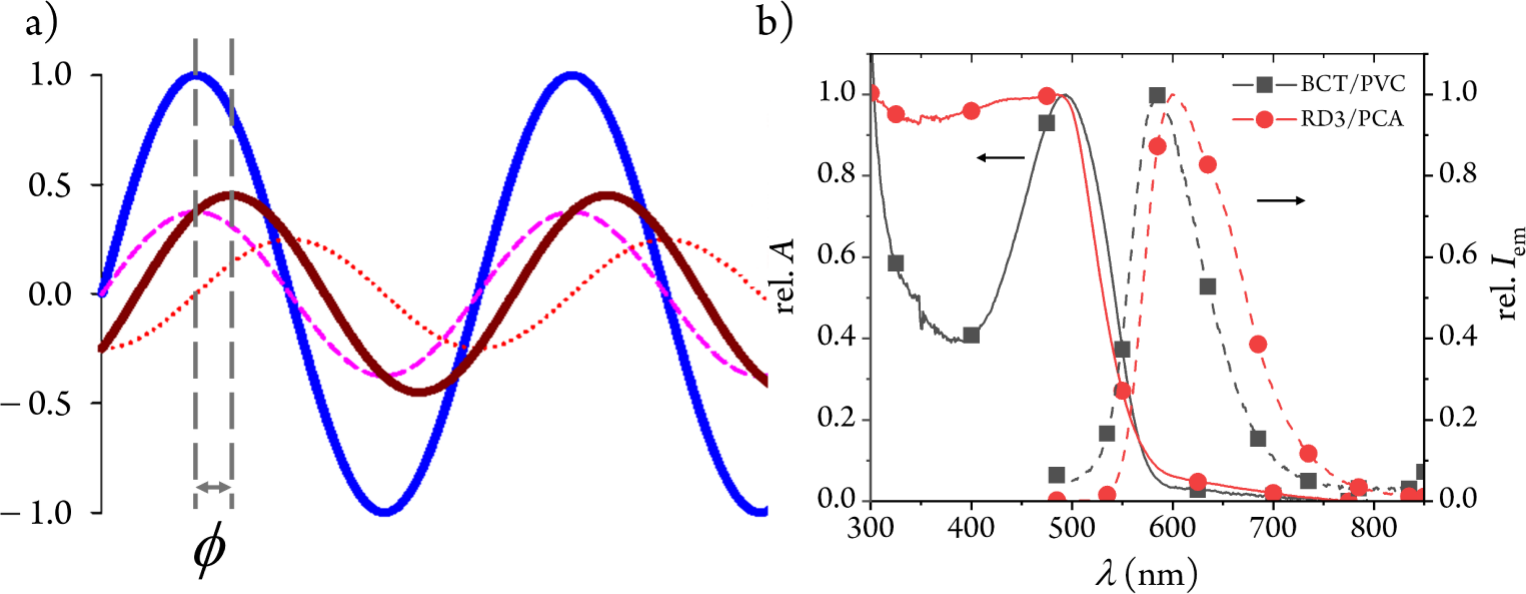
(a) Representation of the DLR principle with sinusoidally modulated excitation blue light (blue solid line) that generates the modulated emissions of the long-τ dye (red dotted line) and the short-τ dye (pink dashed line), and the resulting compound emission signal (brown solid line), all of them with the same frequency *f*. The two dashed vertical lines represent the positions of the maxima of the modulated excitation light and of the modulated compound emission, respectively, so that ϕ is the measured luminescence phase shift. (b) Relative absorption (solid lines) and emission (dashed lines) of the short-τ (BCT/PVC, box solid in black) and long-τ (RD3/PCA, circle solid in red) polymer-supported dyes (see below) with overlapping absorption and emission regions we have used to implement the DLR method.

The variation in the emission intensity ratio in DLR-based sensors occurs when the interaction of the analyte with the indicator dye leads to a variation in its emission intensity. In our case, however, the decrease in luminescence from the indicator dye is not related to any chemical interaction but rather to the blocking of the excitation and emission light passing through the turbid medium (Figure 1).

DLR is insensitive to fluctuations of the light source radiant power and detector aging. However, it has some noticeable drawbacks: it is, like all intensity-based techniques, dependent on the photostability of the dyes or on the presence of light-absorbing materials, leading to the decrease of the radiant flux through the water sample.

### Selection of the Short-τ and Long-τ Dyes

As stated above, the difference between the reference and indicator emission lifetimes should be as large as possible to maximize the effect of the ratio of luminescence intensities on the measured lifetime. At the same time, we aim to use the very same instrumentation designed to measure other water quality parameters (e.g., dissolved oxygen,^22^ pH,^26^ or electrical conductivity^27^) to benefit from the multichannel capabilities of the modern fiber-optic optoelectronic monitors.^28,29^ Therefore, taking into account their photostability and μs-long luminescence lifetimes in the absence of O_2_, a ruthenium­(II) polypyridyl complex was chosen as the long-τ dye. The excited-state lifetime of these dyes can be enhanced by introducing extended π-electron substituents into the ligands.^30^ To this end, the readily accessible tris­(4,7-diphenyl-1,10-phenanthroline)­ruthenium­(II) (RD3) was chosen. However, as ruthenium polypyridyls are significantly quenched by O_2_, RD3 had to be immobilized into a gas-impermeable matrix. Therefore, poly­(ethyl 2-cyanoacrylate) (PCA) was chosen as the support due to its transparency, mechanical properties, ability to dissolve the complex by the monomer, and ease of moldability. Unfortunately, it slowly degrades when submerged in water, so we placed the dye-embedded monolith behind a glass window in the sensor housing.

Ruthenium polypyridyl complexes are also known for their large Stokes shift. This is a desirable feature for luminescence sensing as the excitation and emission can be readily separated using simple optical filters. However, for the DLR-based sensing to work, this large Stokes shift limits the number of usable short-τ organic dyes. The emission of the latter must be nearby or overlap the luminescence of the ruthenium dyes to allow joint collection. To increase the small Stokes shift of fluorophores, conjugated electron-withdrawing and electron-donating substituents must be introduced to provide an additional emissive excited state of the intramolecular charge transfer type.^31–33^ Therefore, after a literature search for a suitable short-τ dye for RD3, BCT (Chart 1) was identified.^34^ We determined the photophysical properties of this single benzene-based fluorophore in 1,2-dichloroethane (DCE) to mimic the environment within the PVC support. The absorption maximum of BCT in this solvent was found to be at 490 nm, and the emission peak was found to be at 596 nm, with an emission lifetime of 6.9 ns (Table 1). When embedded in PVC, its absorption and emission overlap with those of RD3/PCA, fulfilling the requirement for DLR sensing (Figure 2b and Table 1).

**Table 1:** Photophysical Properties of BCT and RD3 in Solution and Immobilized in a Polymer Matrix

Dye/medium^a^	λ_abs_^max^ (nm) (ε_abs_^max^ (L mol^–1^ cm^–1^))^b^	λ_em_^max^ (nm)^b^	τ^c^ (ns)	Φ^d^
BCT/DCE	493 (5470)	596	6.9	0.22^e^
BCT/PVC	486	583	10.5	n.d.
RD3/ACN	463 (33300)	615	178	0.404^f^
RD3/PCA	490	600	4852	n.d.

a DCE: 1,2-dichloroethane; PVC: poly­(vinyl chloride); ACN: acetonitrile; PCA: poly­(ethyl 2-cyanoacrylate).

b Uncertainty: (λ^max^ ± 1) nm; (ε ± 3%) L mol^–1^ cm^–1^.

c Uncertainty: ± 0.5%.

d Emission quantum yield; n.d. = not determined.

e Determined using Rhodamine 6G in EtOH (Φ = 0.94)^35^ as standard; uncertainty: ± 0.02.

f Ref.^30^ in O_2_-free medium; uncertainty: ± 0.005.

### Design and Development of the Turbidity Sensor

To benefit from our multichannel optoelectronic monitor for Ru­(II) polypyridyl-based chemical sensing,^30^ the turbidity terminal was designed to be interrogated through a bifurcated fiber-optic bundle. The backscattering principle at θ = 180° (see above) has been employed to ensure that the emission from both the reference dye and the indicator dye layers can be guided back to the detector (Figure 1). As per the DLR principle, the luminescence intensity of one of the two dyes (indicator) must vary as a function of water turbidity so that the measured phase shift changes. To meet this requirement, the other dye (reference) should be positioned next to the fiber tip to provide a constant emission signal. The indicator dye layer is placed at a distance from the reference layer to allow the sample to lower the emission intensity of the former, depending on the water turbidity. To this end, a two-part sensor terminal was designed to host the dye layers. The top part, called the “head”, holds the reference dye layer and can be adjusted to vary the distance between the reference and indicator dye layers. The bottom part, or “housing”, accommodates the latter and is fitted with a highly polished mirrored surface on the back to reflect the luminescence back to the fiber aperture (Figure 1). Both the reference and indicator layers are sealed behind their respective glass windows.

The reference dye next to the fiber tip might be either the luminescent ruthenium complex or the fluorescent organic dye. The first option would certainly be better for the photostability of the sensor since the more photostable dye is placed where the excitation light has a higher radiant power. However, due to the stronger absorption of RD3 in the blue and the lower emission quantum yield of BCT (Table 1), the RD3 luminescence would be perturbed less by the fluorescence of BCT, leading to a smaller DLR signal variation. Indeed, when we tested this configuration, the emission from the ruthenium complex was hiding any emission from the BCT dye, and no changes with turbidity were detected (data not shown). However, when the position of the dye layers was reversed, a significant change in the measured phase shift could be detected. Therefore, BCT was chosen as the reference dye and placed in a very thin layer at the head, while RD3 played the role of the indicator dye and was positioned in the housing.

### Sensor Tuning and Characterization

After the position of the two dyes was set, their relative concentration was optimized at a fixed distance of 2.0 cm. As the ruthenium complex was placed further away from the fiber tip, a concentration of 1.2 mg mL^–1^ in the cyanoacrylate monomer solution (the highest possible one) was used. This high concentration ensures that enough emission of the ruthenium monolith reaches the fiber tip. Then, five different concentrations of BCT in PVC within the 0.015–0.3 mmol L^–1^ range were tested by placing the assembled sensor terminal in clear water (i.e., filtered through a 0.22 μm membrane). In this way, the lowest BCT concentration showed a ∼26° phase shift (Figure S3). Considering that the unperturbed luminescence phase shift of the monolith is 50° at a modulation frequency of 39 kHz (eq 1; τ = 4852 ns, Table 1), we selected the lowest BCT concentration as the optimum one because it would allow us to tune the optical path length in the next step.

Having set the concentration of the dye layers, the optical path length of the indicator layer to the fiber tip must be optimized to tune the turbidity measuring range. The separation between the reference and indicator layers and the water turbidity level will lead to a stronger or weaker perturbation of the long-τ dye emission by the short-τ dye fluorescence. To this end, three heads with 1.0, 1.5, and 2.0 cm optical path lengths were 3D printed (Figure 1 right). The path length effect was initially estimated by calculating the ratio of the emission intensities (*A*_S_/*A*_L_, amplitudes of the emission sine waves provided by the optoelectronic instrument) from the luminescent monolith and the fluorophore layer. The emission intensity from the RD3-embedded monolith was obtained by placing the sensor terminal without the BCT layer into 0, 50, 100, and 250 NTU standards. The emission intensity from the fluorescent layer was collected by removing the housing from the head and measuring the signal under water. The resulting ratio (Figure 3a) shows that the emission intensity of the monolith is higher than that of the BCT layer for the 1 cm optical path length regardless of the water turbidity, while the emission of the BCT layer is always stronger for the 2 cm path length. The intermediate path length yields roughly equal emission intensities at 0 NTU, but their ratio increases with the water turbidity. The slope of the *A*_S_/*A*_L_ vs the water turbidity plot also changes significantly, with the 2 cm path length showing the highest value. This result is to be expected as the emission intensity from the monolith is the denominator in the ratio, so its variations have a higher impact when the initial emission ratio is above 1 (Figure 3a).

**Figure 3:**
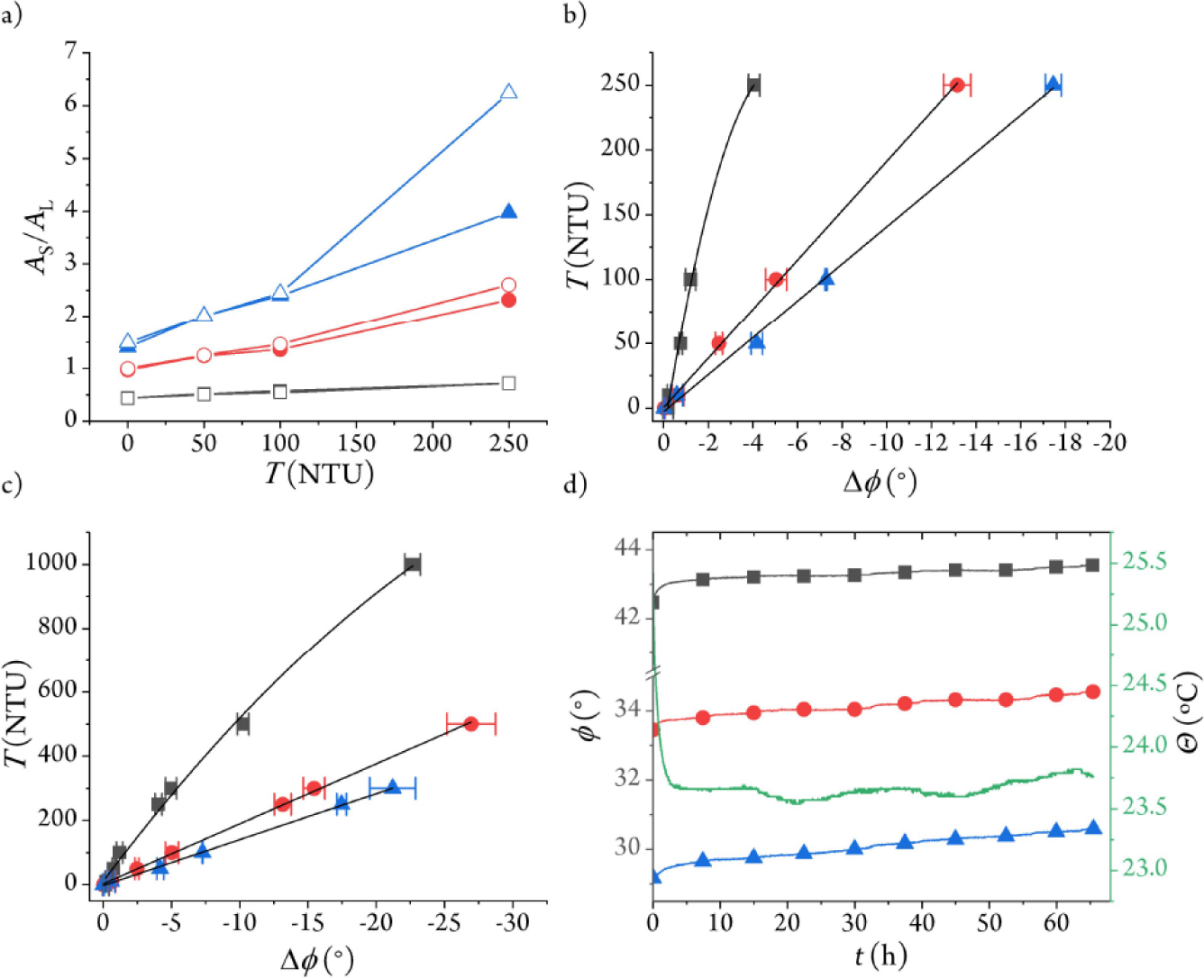
Calibration plots of the 1 cm sensor (box solid in black); 1.5 cm sensor (circle solid in red); 2 cm sensor (triangle up solid in blue). (a) Emission intensity ratio of the BCT reference (*A*_S_) and the RD3 indicator (*A*_L_) layers at different distances and turbidity values, monitored through the 590 nm broadband interference filter (solid symbols) or the (615 ± 10) nm bandpass interference filter (open symbols) at 20 °C. (b) Phase shift differences (excursion) from 0 NTU; the solid lines represent the least-squares fits: 1 cm sensor, Τ = – 19 – 108Δϕ – 10­(Δϕ)^2^ (*R* ^2^ = 0.998); 1.5 cm sensor, Τ = – 1 – 19.1Δϕ (*R* ^2^ = 0.999); 2 cm sensor, Τ = – 2 – 14.3Δϕ (*R* ^2^ = 0.998). (c) Phase shift differences of the sensor terminals with turbidity values higher than 250 NTU; the solid lines represent the least-squares fits: 1 cm sensor (0–1000 NTU), Τ = 10 – 57Δϕ – 0.6­(Δϕ)^2^ (*R* ^2^ = 0.996); 1.5 cm sensor (0–500 NTU), Τ = 3 – 18.7Δϕ (*R* ^2^ = 0.999); 2 cm sensor (0–300 NTU), Τ = – 3 – 14.3Δϕ (*R* ^2^ = 0.999). (d) Sensor stability over 65 h at 23.5 °C in clear water.

The turbidity sensor terminals with different path lengths were calibrated up to 250 NTU, and the corresponding phase shift excursions were calculated thereof (Figure 3b and S4; the phase shift data have been placed on the *x*-axis to facilitate the calibration transfer to the optoelectronic measuring system). Using the excursion instead of the absolute ϕ value as the analytical signal eliminates signal variations resulting from the handcrafted fluorescent and luminescent dye layers. The 0–250 NTU turbidity range was chosen because it encompasses the typical turbidity values of surface waters in Spain.^36–38^ As predicted by the emission intensity ratios, the 2 cm path length sensor showed the highest sensitivity (0.07°/NTU), boasting over 18° phase shift difference between 0 and 250 NTU. The 1.5 cm sensor (0.05°/NTU) shows ∼10° phase shift excursion at the highest turbidity level tested, while the 1 cm sensor (0.02°/NTU) displays the lowest phase shift difference (∼4°, Figure 3b).

Figure 3 demonstrates that the optical path length of the sensor terminal can readily be used to tune the sensor to the turbidity range of interest. Actually, an increase in the distance between the reference and indicator layers leads to higher sensitivity at the expense of a narrower turbidity range. Nevertheless, the sensor is not limited to the 250 NTU range, and higher values were tested to demonstrate this fact (Figure 3c). Based on our results, the 2 cm path length sensor is best suited for 0–300 NTU, while the 1.5 and 1 cm sensors respond up to at least 500 and 1000 NTU, respectively. However, as our sensor’s raw signal is theoretically limited to the 0°–50° phase shift range (see above), the 2 cm sensor is close to its high limit of detection (LOD) at 300 NTU due to the associated absolute phase shift of ∼4°. Using the fitting functions, high LODs of 560 and 3050 NTU can be estimated for the 1.5 and 1 cm path length terminals.

The low LOD for the 2, 1.5, and 1 cm sensors are 0.1, 0.2, and 0.5 NTU, respectively, calculated using the fitting functions and three times the peak-to-peak noise of the phase shift at 0 NTU (0.02°).^39^ According to our measurements (Figure 3b), all sensors show an accuracy of 1 NTU in the 10–250 NTU range, which is limited by the accuracy of the formazin standard (1 NTU).^6^ Nevertheless, the 1.5 cm and 2 cm sensors exhibit higher accuracy (0.4 and 0.3 NTU, respectively) in the 0–10 NTU range due to the higher accuracy of the standard (0.1 NTU). Taking into account that the integration time of the measurement is set at 2 s (Supporting Information), the response time cannot be lower than this value. Therefore, since the DLR signal changes instantaneously, the response time is only limited by the instrument configuration.

The sensors were also tested for 3 months to gauge the measuring repeatability after complete disassembly and reassembly of the terminals between the measurement dates (Figure S5). As expected under those conditions, the repeatability of the phase shift excursion at 10 NTU is poor—namely, 49% for the 1.5 cm sensor and 29% for the 2 cm sensor—and averages 5–18% for higher NTU values and for the 1 cm sensor. Nevertheless, when the sensor readings are taken without disassembling the terminal (but removed from the sample to clean it between samples of different turbidity), repeatability improves to an average of 5%, 14%, and 11% for the 1, 1.5, and 2 cm sensors, respectively, across their measurement range (Figure 3b). This improvement shows that the sensor terminals must be manufactured with all optical components fixed in place as the underlying DLR principle is a luminescence intensity-based technique, and any geometrical changes lead to turbidity-unrelated phase shift variations. Obviously, repeatability could be further improved by installing the sensor terminal in the sample holder and keeping it in place for continuous measurements, as has been carried out for the in situ testing.

A comparison between the figures of merit of the luminescence-based and some commercially available turbidity sensors is shown in Table S1. While the commercial sensors display a somewhat extended measuring range (up to 4000 NTU), our 1 cm sensor terminal only reaches ca. 3050 NTU, although this range can readily be increased by shortening the optical path length. Unfortunately, the specifications for sensor repeatability have not been reported by the various manufacturers.

Since the sensor principle relies on the ratio of two emission intensities, it is paramount to test the stability of the reference and indicator layers in the sensor terminal. As the ruthenium dye is known to be highly photostable and its layer is located further away from the excitation light, only the stability of the BCT layer was tested through an accelerated photobleaching test. To this end, the reference layer was irradiated for 15-s illumination periods interspersed with a 10-s dark interval for 90 h (Figure S6). Under these conditions, the layer receives a 54-fold higher photon flux at 470 nm than under regular operating conditions. The initial emission intensity drops to about 30% after 90 h of illumination.

Then, three sensor terminals were placed in clear water for ca. 3 days (Figure 3d), setting the regular conditions of 2-s illumination time every 3 min. The phase shift readings of the 1 cm sensor are the most stable ones, with just a 0.46° increase over the measurement time and a 0.007°/h drift. The 1.5 cm sensor shows a growth of 0.78° over the same period, equal to a 0.012°/h drift, while the 2 cm sensor is the least stable one, with an overall increase of 1.06° (0.016°/h drift). These values correspond to a signal increase of just 0.23 NTU/h. The positive slope results from the decrease of the *A*_S_/*A*_L_ ratio (eq 2) over time due to the slow photobleaching of the fluorescent layer (*A*_S_). The latter is subject to a higher radiant flux than the luminescent monolith, and the BCT organic dye is less photostable than the Ru­(II) complex. Therefore, the highest stability of the 1 cm path length sensor is the consequence of the lowest contribution of the fluorophore emission to its signal (see above).

Since the ruthenium complexes, unlike short-lived fluorophores, are known to be influenced by both temperature and dissolved O_2_,^30,40^ the sensor’s cross-sensitivity to these parameters must be tested. For the temperature tests, the sensor manifold, the sample container, and the turbidity standards were equilibrated at the target temperature for 24 h in the thermostatic chamber before starting the experiment. As expected, the response was found to depend on the water temperature (Figure 4a–f). The absolute phase shift excursion of the 1 cm sensor decreases as the temperature rises from 8 to 15 °C and increases again at temperatures above 25 °C. However, the excursion seems to be independent of the tested temperatures for the 2 cm sensor. The 1.5 cm sensor displays mixed behavior, decreasing its phase shift excursion above 15 °C.

**Figure 4:**
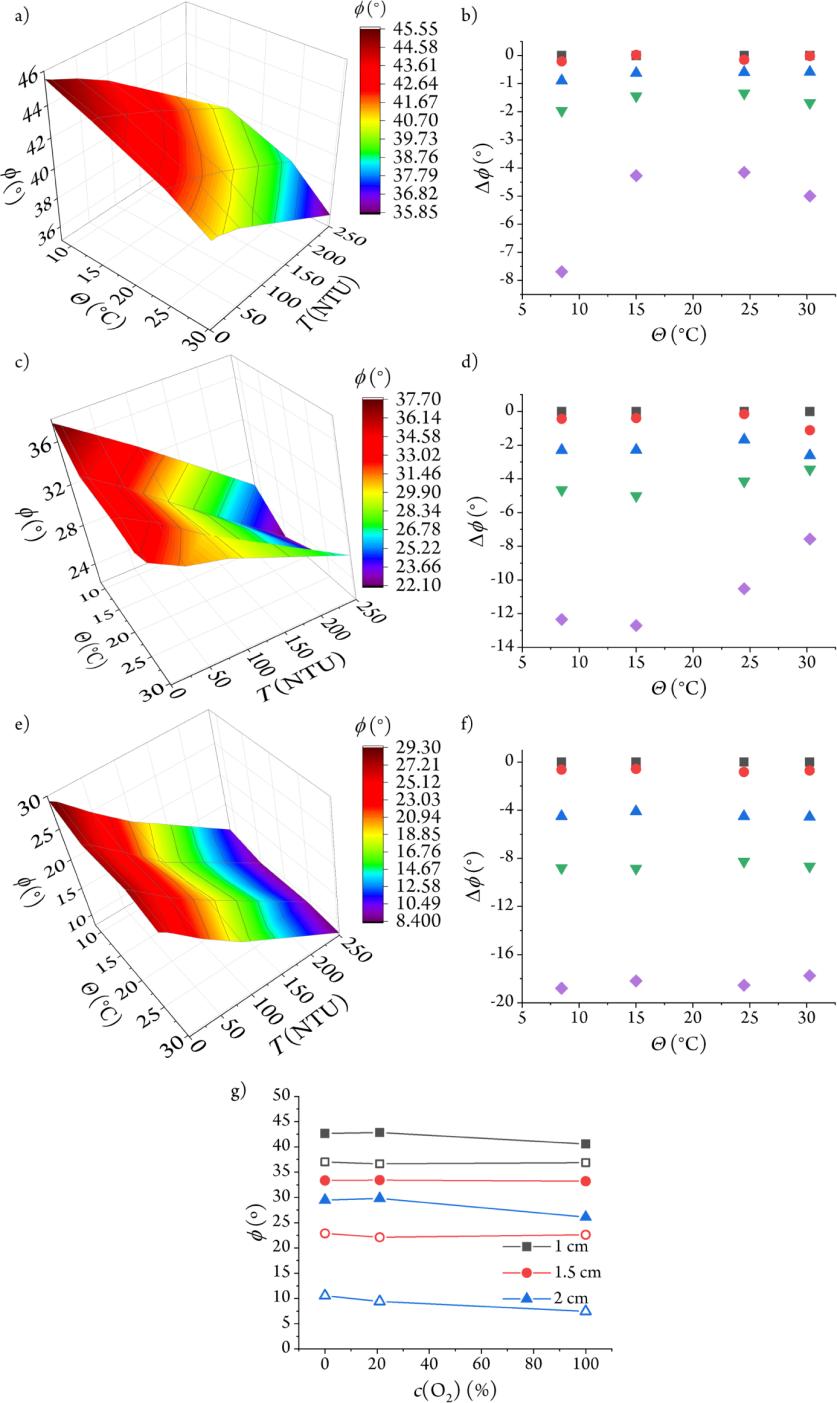
Effect of temperature on the 1 cm (a), 1.5 cm (c), and 2 cm (e) path length sensors and the corresponding phase shift excursions from 0 NTU for the 1 cm (b), 1.5 cm (d), and 2 cm (f) sensor. The tested turbidity values are 0 NTU (box solid in black), 10 NTU (circle solid in red), 50 NTU (triangle up solid in blue), 100 NTU (triangle down solid in green), and 250 NTU (tilted square solid in violet). Sensor response as a function of O_2_ concentration in 0 NTU (filled symbols) and 250 NTU (hollow symbols) solutions (g).

These temperature effects arise from the intricate interplay between the optical path length and the *A*_S_/*A*_L_ ratio. While the emission of the reference dye is constant over the tested temperature range (Figure S7), the indicator monolith shows significant changes in both its luminescence intensity and lifetime.^40^ The temperature effect is further complicated by the turbidity-induced partial blocking of the ruthenium monolith emission and its influence on the *A*_S_/*A*_L_ ratio (Figure 3a). To account for the change in the analytical signal due to temperature variations, a 3D calibration table (from Figure 4a,c and e) is introduced into the control software of the optoelectronic monitor.^41^

Regarding dissolved O_2_, we were not expecting a large effect since both the reference and indicator dyes are embedded into gas-impermeable polymer supports. Furthermore, the short emission lifetime of the immobilized BCT fluorophore (10.5 ns) avoids the O_2_ interference. The longer-lived RD3 indicator^42^ displays a small O_2_ effect on the outermost layer of the monolith and, therefore, on the turbidity sensor response (Figure 4g and Table S2).

Another major source of interference is the presence of air bubbles in the optical path of the sensor. They provoke a decrease in both the blue excitation light and the red emission of the RD3-doped monolith. This leads to an artificially lowered phase shift and grossly overestimated turbidity as the relative contribution of the emission from the reference dye layer increases (Figure S8). Therefore, in order to obtain meaningful turbidity measurements, care must be taken to keep the sample under laminar flow, not only to prevent air bubble formation but also to provide stable readings. Furthermore, the water flow avoids the settlement of suspended particles when measuring in situ.

Regarding the potential effect of ambient light, we have checked that laboratory illumination has no influence on turbidity measurements. However, direct sunlight falling into the mirrored surface of the sensor terminal placed in low-turbidity waters might be reflected into the optical fiber, thereby saturating the detector and preventing meaningful measurements.

### Effect of Waterborne Chlorophyll

For in situ water monitoring, the effect of chlorophyll (Chl) within algae or cyanobacteria cannot be overlooked because of its absorption in the blue and red emission features. Commercial turbidity sensors circumvent this problem by using near-infrared light sources, where chlorophylls do not absorb or emit.^8^ However, to obtain the maximum emission intensity from the immobilized RD3, a 470 nm LED was chosen as the excitation light source. Chl *a* displays absorption at 417 and 659 nm and fluoresces at 671 nm in methanol^43^ (684 nm in algae),^44^ while Chl *b* shows slightly blueshifted values. The presence of Chl would lead to an artificial decrease in the emission intensity of the indicator layer owing to its absorption in the blue. Furthermore, its ns-lived emission^45,46^ leads to an additional contributor to the DLR scheme. Therefore, the overall effect of Chl will be a lowered phase shift, leading to overestimation of the sample turbidity (Figure 5).

**Figure 5:**
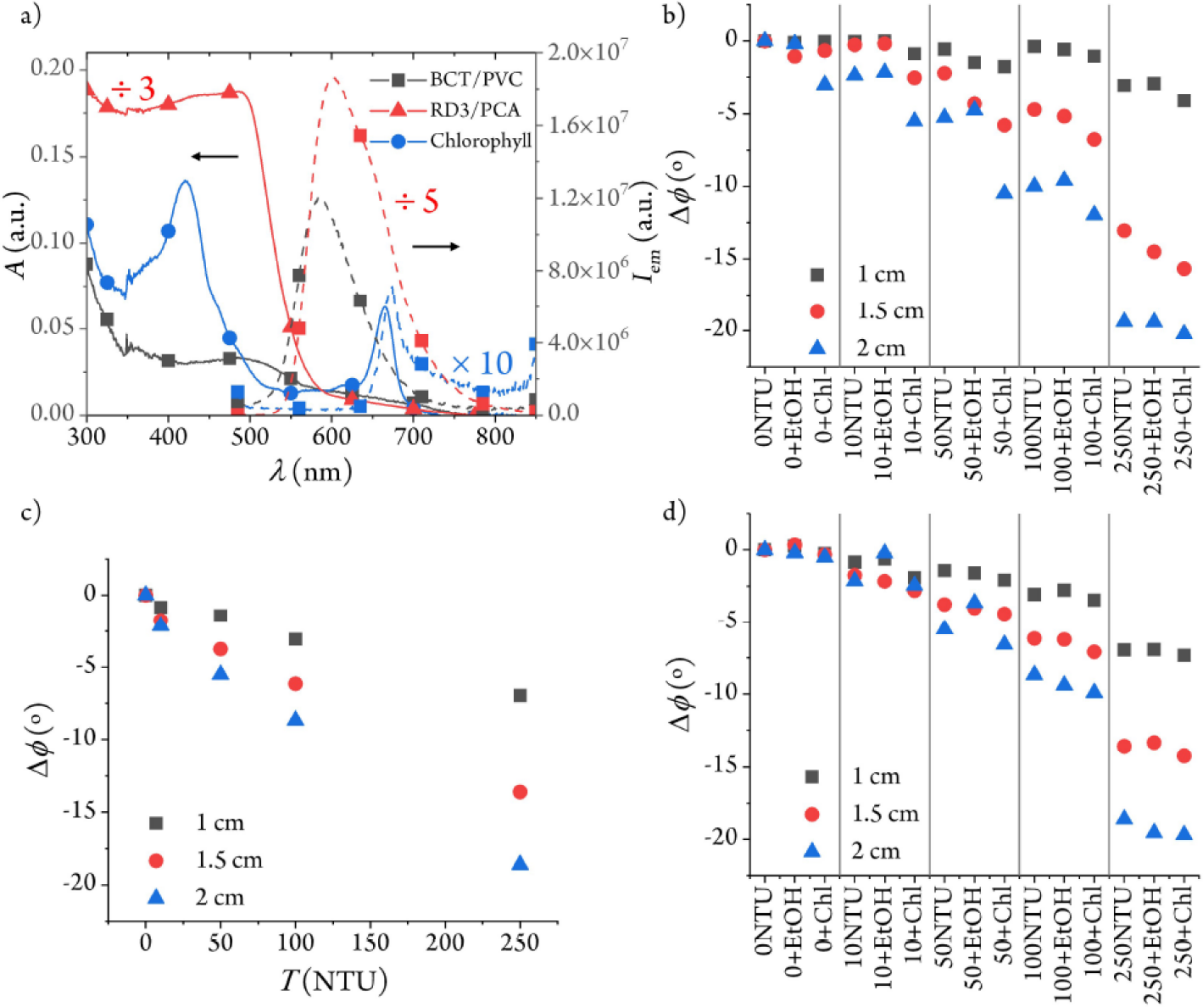
(a) Absorption and emission spectra of 1.2 mg g^–1^ RD3 in PCA, 0.06 mg g^–1^ BCT in PVC, and 0.35 μg mL^–1^ chlorophyll in 10% EtOH–H_2_O (v/v) (see Supporting Information). (b) Response of the sensors to different turbidity levels in terms of phase shift excursions from 0 NTU. Each of the three measurement sets corresponds to a particular turbidity standard, the same turbidity standard with 10% EtOH, and the same turbidity standard with a 0.33 μg mL^–1^ chlorophyll extract in 10% EtOH–H_2_O (v/v). c) Response of the 1, 1.5, and 2 cm turbidity sensors to different NTU values at 25 °C using the 615 nm interference filter in front of the detector instead of the 590 nm broadband filter. (d) Same as (b) with the 615 nm interference filter in front of the detector.

A Chl extract was used instead of whole algae cells to exclude turbidity effects that were unrelated to the pigment. As depicted in Figure 5a, our Chl extract in aqueous solution shows non-negligible absorption at 470 nm and an additional absorption band at 664 nm. While its emission band at 674 nm does not overlap with the BCT fluorescence, the broad luminescence from RD3 extends into the region of Chl emission. Moreover, we have measured the fluorescence lifetime of the Chl extract was 3 ns. Therefore, the pigment fulfills the DLR requirements and would contribute to decreasing the phase shift signal.

To investigate the effect of Chl on the sensor, a 3.33 μg mL^–1^ EtOH extract was mixed with the formazin standard solutions, and their turbidity was measured. The final concentration of Chl (0.33 μg mL^–1^) is over 200 times higher than the typical values in surface waters (∼1.5 μg L^–1^ to 3.0 μg L^–1^).^47^ However, as the pigment is not soluble in water, the alcoholic solution of chlorophyll is needed to incorporate the pigment into the aqueous formazin standards. Unfortunately, Chl forms aggregates when the water content exceeds 80% in methanol or 60% in ethanol.^48^ As we are unsure about the effect of the high organic solvent content on the scattering properties of the formazin standards, our approach was to keep the ethanol content as low as possible (i.e., 10% v/v). The resulting Chl aggregates only show 20% of the emission of a solution of the same concentration in pure EtOH (Figure S9). Therefore, we estimate that the contribution of the 330 μg L^–1^ Chl solution to the *A*_S_/*A*_L_ emission ratio (eq 2) may be closer to that of a 70 μg L^–1^ nonaggregated Chl solution. This concentration is closer to those expected during algae blooms in summer or in eutrophicated waters.^49,50^

Figure 5b shows the effect of 0.33 μg mL^–1^ chlorophyll on the different sensors. Since Chl has a short emission lifetime, it should interfere mostly with the sensor when the emission intensity from the indicator layer is similar to the emission intensity from the reference layer. As expected from the predominance of the ruthenium emission, the 1 cm sensor is not significantly affected by the addition of the pigment at low NTU values, but the phase shift excursion changes by ∼1° at 250 NTU (∼77 NTU). For the 1.5 cm sensor, the effect of chlorophyll increases due to the higher amount of pigment in the optical path and, therefore, the presence of short-lived fluorescence. At the same time, a higher Chl concentration leads to more blue light absorption and less ruthenium emission. Subtracting the contribution of EtOH from the phase shift excursion, the 1.5 cm sensor shows the highest interference at 10 NTU (Δϕ ∼2.4° or 43 NTU), while the 2 cm sensor displays it at 50 NTU (Δϕ ∼5.2° or 69 NTU). At high turbidity levels, the emission from RD3 is mostly blocked so that the effect of chlorophyll on the emission phase shift is negligible since the fluorescence from the reference indicator layer predominates.

One possible way to reduce the influence of Chl could be to replace the 590 nm broadband interference filter by a 615 nm narrow bandpass filter in front of the detector to remove the narrow emission of Chl at 674 nm. However, as the emission of both RD3 and BCT extends beyond 625 nm, some part of their emission will also be blocked, and the signal will decrease. The latter can be compensated for by increasing the detector gain. When the chlorophyll interference experiments were repeated with the new optical filter, the sensitivity of both 1 and 1.5 cm sensors increased (Figure 5c). The highest interference of Chl is now found at 100 NTU with the 2 cm sensor (Δϕ ∼ 1.2° or only 17 NTU, Figure 5d). This result shows that the Chl effect can be readily removed by simple instrument tuning.

### Turbidity Sensor In Situ Testing

To test the sensor out of a controlled environment, a cul-de-sac water supply pipe (11.4 cm or 4.5″ diameter) was found at Complutense University of Madrid and used as the testing site (Figure 6a). Since it belongs to an urban water infrastructure, we only used the 2 cm sensor head to provide maximum sensitivity. Therefore, three of these sensor terminals (Figure 1) were introduced into a custom-made PVC flow cell (Figure 6b,c). The latter was fitted with 7 mm i.d. water inlet and outlet and connected to a 3/8 valve on the pipe via 8 × 12 mm silicone tubing. The sensor terminals were positioned on an adjustable stainless steel mesh platform inside the flow cell above the water inlet so that the incoming water flow did not directly enter the optical paths. The rate was set to ∼140 mL min^–1^ to ensure a laminar flow (Reynolds number, Re = 32), and the water temperature was monitored in the flow cell. The results of the 11-day monitoring are shown in Figure 6d and S10.

**Figure 6:**
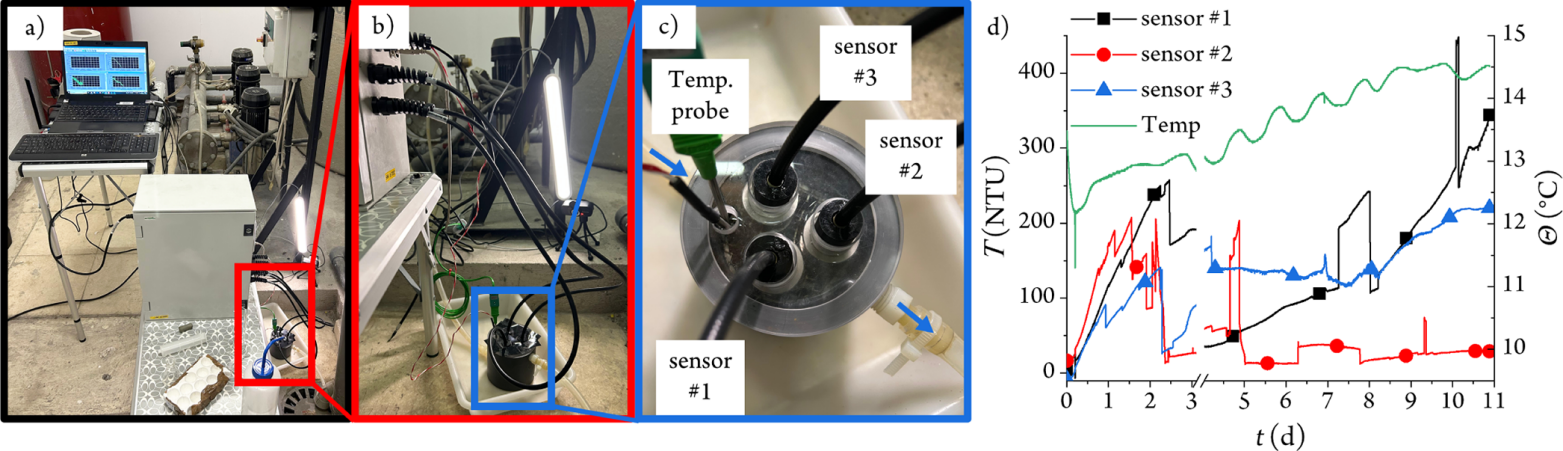
(a) Overview of the measuring site showing (counterclockwise) the cul-de-sac water pipe, the laptop control unit, the optoelectronic fiber-optic monitoring unit, and the sample flow cell. The water pressure pumps are never used due to sufficient pressure. (b) Close-up view of the optical fibers, the temperature probe, and the flow cell. (c) Top view of the flow cell with the sensor manifold; the blue arrows indicate the water flow direction. (d) Response of the three sensors to turbidity for 11 days. The measurement was reset on day 4 after a power outage on day 3.

Prior to the start and after the run, the three sensors were calibrated using standard turbidity solutions to verify that no change in sensitivity had occurred (Figure S11). After the start of the continuous measurement, an increase in turbidity was recorded as the water flow released the accumulated sediments in the cul-de-sac pipe (see below). After ca. 2 days of rising, the turbidity unexpectedly dropped over the course of a few hours in all three sensors, alongside some turbidity spikes. We noticed that, at this time of the measurement, the water flow had decreased considerably due to partial obstruction of the valve by suspended particles, but we did not change it. The reduced flow rate could possibly have led to sedimentation of the latter inside the flow cell, with a concomitant lowering of the turbidity. When data collection resumed after the power outage on day 4, sensor #2 did not record any important turbidity changes, its steady readings being only perturbed by occasional air bubbles. Sensor #3 measurements also remained stable within the following 5 days but above those of sensor #2. This is probably due to particles settling on the monolith glass window of this sensor due to its position in the flow cell. At present, we have no explanation for the continuous rise in the turbidity readings of sensor #1, which were only perturbed by sporadic air bubbles. The low turbidity measurements obtained right at the start of the run for all three sensors were probably due to the smooth sampling of clear water from the upper layer in the pipe.

When the sensors were taken out of the flow cell after the 11-day run, rust particles were found onto the monolith glass window of sensor #3, less so on the other sensors. Moreover, the water flow had decreased to 14 mL min^–1^, and a persistent reddish-brown residue was found to be stuck on all surfaces. Then, the inlet valve was fully opened, and a sample of water was taken to confirm the presence of rust particles (Figure S12). To mitigate these interferences, a slightly higher flow rate should be used together with a sampling point placed horizontally on the pipe to prevent air pockets.

## Conclusions

A new type of backscattering water turbidity sensor using the luminescence-based dual-lifetime referencing (DLR) scheme has been developed. The latter allows sensor interrogation with current commercial optoelectronics for dissolved O_2_ luminescent sensing. The unique design of the sensor terminal enables the user to tune the turbidity range for optimum sensitivity depending on the actual operation conditions. The sensor is dissolved oxygen- and waterborne chlorophyll-proof and only requires temperature correction. Selecting a more photostable reference fluorophore and replacing handcrafting elements with precision machining would lead to further improvements in the sensor’s stability and accuracy. The sensor has been shown to perform effectively in real-world environments during continuous in situ measurements of water turbidity in the 20–300 NTU range.

## Supplementary Material



## Notes

Additional syntheses, chemicals and reagents, instrumentation, procedures and additional graphs (PDF)
