# Supplementary material for: Luminescence Lifetime-Based Sensing of Water Turbidity
Source: ACS Sens. 2025 May 7;10(5):3763–73. doi: 10.1021/acssensors.5c00849 (PMC12123664; doi:10.1021/acssensors.5c00849)
Supplement: Supplementary file 1 [file se5c00849_si_001.pdf]

Supporting Information

Luminescence lifetime-based sensing of water turbidity

Ya Jie Knöbl, Iman Nakhli, María del Mar Darder, Guillermo Orellana*

Chemical Optosensors & Applied Photochemistry Group (GSOLFA), Dpmt. of Organic Chemistry, Faculty of Chemistry, Complutense University of Madrid, 28040 Madrid, Spain.

Chemicals and Materials

All organic solvents used (Analytical or HPLC grade) were supplied by Merck (Darmstadt, Germany), Acros Organics (Geel, Belgium), or VWR (Radnor, PA, US). Reagents for synthesis were purchased from TCI (Tokyo, Japan), Fluorochem (Derbyshire, UK), Merck, Fluka (Büchs, Switzerland), or Acros. NMR solvents were from Merck, Acros or VWR and were of >98.6% isotopic purity. Type I water was obtained from a Millipore Direct-Q® 3UV purification system. Formazin turbidity standards (1000 NTU, 500 NTU and 100 NTU) were purchased either from VWR or Merck. Other values were obtained by dilution with type I water filtered through 0.22 µm filter.[1] 3D printing filament (PET-G) was provided by BCN3D (Barcelona, Spain). Dinitrogen, argon, dioxygen and synthetic air of +99.995% purity from cylinders were supplied by Carburos Metálicos (Madrid, Spain). Gas mixtures were prepared with a PID Eng&Tech (Alcobendas, Madrid, Spain) electronic mass flow-controlled mixing unit.

Synthesis of luminescent dyes

Tris(4,7-diphenyl-1,10-phenanthroline)ruthenium(II) bis(hexafluorophosphate) ($[Ru(dpp)_3](PF_6)_2$)

The synthesis of $[Ru(dpp)_3](PF_6)_2$ follows a slightly modified preparation of that described in the literature.[2] $RuCl_3$ hydrate (55.5 mg, ca. 1 mmol) and 4,7-diphenyl-1,10-phenanthroline (120.0 mg, 3.3 mmol) are placed in a two-necked round bottom flask and flushed with argon. Then, 7 mL of ethylene glycol is added and the mixture deoxygenated for 20 min using argon. After increasing the temperature over 3 h until reflux, the reaction is further heated for 24 h before allowing cooling down to room temperature. Then, 1.5 mL of a saturated aqueous solution of ammonium hexafluorophosphate is added dropwise to the reaction solution and the resulting suspension cooled at 4 °C for 2 h. The orange precipitate is collected via vacuum filtration and dried under vacuum overnight. The NMR matches the literature spectrum.[2] Yield: 110 mg (93%).

Dimethyl 2,5-bis(cyclohexylamino)terephthalate (BCT)

The synthesis of BCT follows a modified preparation to that described by Kim et al.[3] To a 25 mL round-bottom flask 228 mg of dimethyl 1,4-cyclohexanedione-2,5-dicarboxylate (1.0 mmol) is added, together with 10 mL absolute EtOH and 1 mL glacial acetic acid (17.5 mmol). Cyclohexylamine (0.36 mL, 3.1 mmol) is then added dropwise to the solution and the reaction is heated to reflux. After 18 h, the reaction is cooled to room temperature and the mixture is filtered. The separated solid is washed with absolute EtOH and dried under vacuum overnight. Yield: 264 mg (68%).

^{1}H NMR (300 MHz, THF-d_8) δ/ppm: 7.31 (s, 2H), 6.93 (d, J = 8.4 Hz, 1H), 3.82 (s, 6H), 3.36 (d, J = 7.9 Hz, 2H), 2.47 (s, 4H), 2.45 – 2.39 (m, 2H), 2.02 – 1.95 (m, 4H), 1.33 (dt, J = 19.7, 11.1 Hz, 11H).

Photobleaching test

The BCT membrane (15 μmol L^{-1}) is placed at the common tip of the optical fiber and illuminated using the LED in the optoelectronic unit for 15 s every 25 s. The area between 590 nm and 608 nm is monitored with an OceanOptics Flame spectrometer (Orlando, FL, US) through an OG590 filter and used for the calculation of photodegradation. This setup leads to a 54-fold illumination photon flux compared to that of the regular experimental setting.

Extraction of waterborne chlorophyll

To obtain a chlorophyll stock solution, 750 mg chlorophyll (C0780, TCI) was suspended in 120 mL absolute EtOH. This suspension was sonicated for 1 min before stirring at room temperature for 30 min. The slurry was then vacuum filtered through a Büchner funnel (the filter cake was not washed) and the filtrate used without further purification for the preparation of the formazin + Chl solutions after determining its concentration to be 3.33 mg mL^{-1} and 3.35 mg mL^{-1} for two different preparations, respectively, using UV-VIS spectrophotometry.[4]

Spectroscopic and photophysical measurements equipment

^{1}H-NMR spectra were recorded on a Bruker AVIII HD 300 MHz BACS-60 instrument (UCM NMR Central Instrumentation Facilities).

Spectroscopic measurements of the dye solutions and layers were performed at (298 ± 2) K, contained in Suprasil® cells with a 1 cm pathlength (Hellma, Müllheim, Germany). UV-VIS absorption spectra were recorded with a Varian Cary 3Bio spectrophotometer (Palo Alto, CA, US). Luminescence spectra corrected for the instrument response were measured with a Horiba Fluoromax-4 spectrofluorometer (Kyoto, Japan). For emission measurements, the layers were placed at 135° relative to the emission plane to avoid contamination by the scattered excitation light.

Emission lifetimes (τ) were determined by single photon timing (SPT), using an Edinburgh Instruments FL-980 TCSPC spectrometer (Glasgow, UK) equipped with a Horiba NanoLed-470LH blue laser diode (463 nm, <1 ns FWHM pulses, 100 kHz) as the light source, a double monochromator and a red-sensitive Hamamatsu R928P photomultiplier detector (Hamamatsu, Japan), thermoelectrically cooled at −21 °C. Luminescence phase shifts were measured with a dedicated 4-channel optoelectronic device (Optosen®),[2] which is used to simultaneously interrogate, via bifurcated fiberoptic bundles (Rockwell Automation Allen-Bradley 43GR-FBS25SL, Diegem, Belgium), up to four turbidity sensors.[5,6] The excitation source is a high-intensity 470-nm LED digitally modulated at 39 kHz, the emission of which is passed through a 470-nm wide bandpass interference filter. The emission from the sensitive terminal at the common end is monitored through a 590-nm center wavelength broadband interference filter with a red-sensitive Hamamatsu miniature photosensor module. Each measurement is averaged over a 20-point boxcar with an integration time of 2 s. Under standard conditions, the Optosen® device provides a resolution of 0.02° phase shift.

Turbidity measurements setup

For the turbidity experiment, three housing terminals each one equipped with a head of a different optical pathlength were placed into a home-made flat-bottomed glass container. The container is wrapped in black tape, placed into a thermostatic chamber (VWR INCU-Line 150R Premium) and filled with water sample (200 mL). The latter is kept at laminar flow (88 mL min^{-1}) using a 12-V mini peristaltic pump (G528) and external 2.0 mm i.d. poly(tetrafluoroethylene) tubing. Once the sensor has been temperature-equilibrated, five measurement points are taken with an interval of 3 min between each measurement point (2 s illumination time per 3 min). The turbidity solution is then replaced with the next solution. Temperature of the turbidity solution is monitored with a type K thermocouple (USB-TC01) from National Instruments (Austin, TX, US).

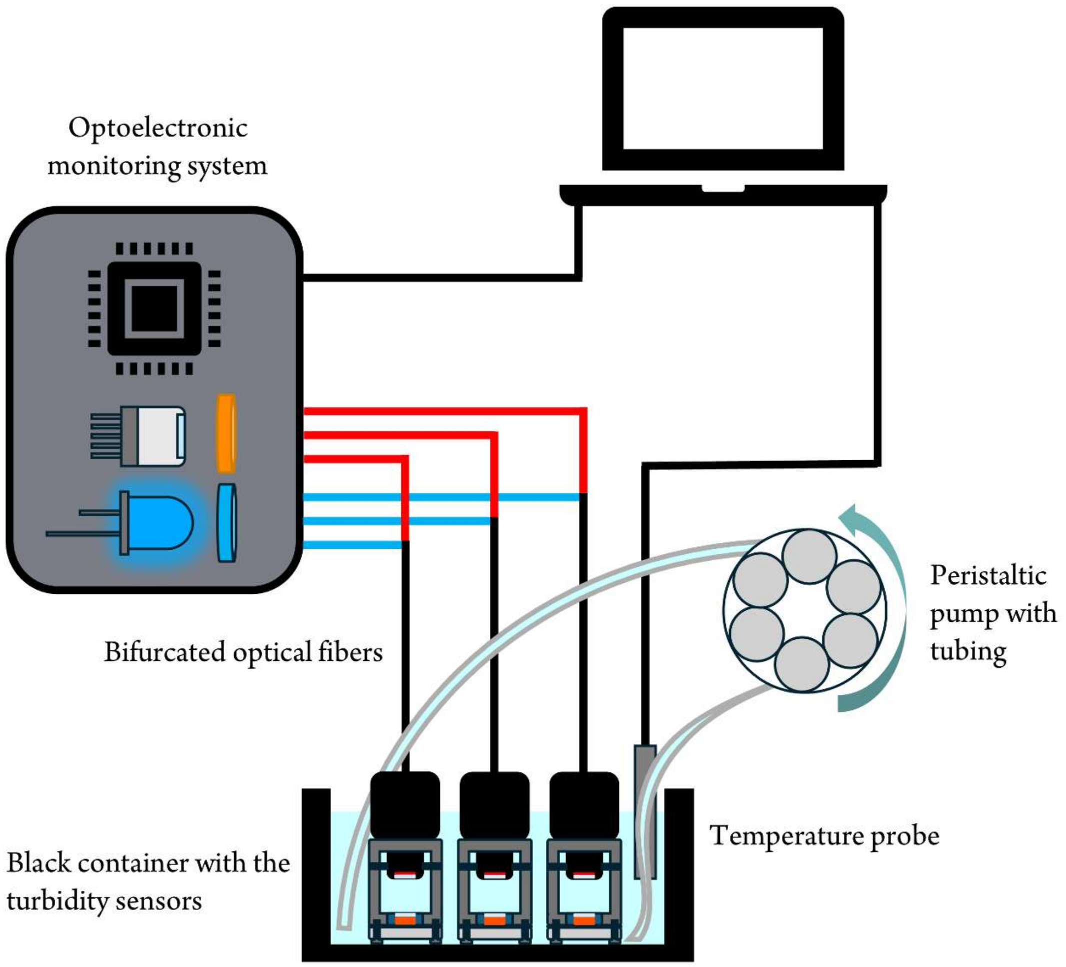

Figure S1. Scheme of the setup for turbidity measurements.

To measure the oxygen effect, synthetic air, dinitrogen or dioxygen were introduced into either clear water or 250 NTU formazin solution using a sparger for at least 30 min. The gas-saturated water is then transferred with a cannula to a closed vessel containing the sensor terminal for the measurement.

For the chlorophyll interference studies, three solutions per turbidity level were prepared. The first solution was the reference solution with the sought NTU value after dilution with water; the second solution was obtained by dilution of the turbidity standard with water and 20 mL EtOH (10% v/v of the final volume); the third solution contained 20 mL of the ethanolic chlorophyll extract, to which water and the corresponding volume of 1000 NTU (or 100 NTU) formazin solution were added.

Table S1. Specifications of some commercial turbidity sensors for environmental monitoring (from the manufacturer's brochure).

	Measuring Technique	Measuring range	Accuracy	Response time (s)
**HACH** Solitax sc Turbidity-Suspended Solids Sensor	Infrared Duo scattered light technique for color-independent turbidity measurement in accordance with DIN EN 27027	0.001 – 4000 FTU/NTU	(up to 1000 FTU/NTU): without calibration < 5 % of the measured value ± 0.01 FNU/NTU with calibration < 1 % of the measured value ± 0.01 FNU/NTU	1 s < t_{90} < 300 s (adjustable)
**WTW** VisoTurb® 700 IQ	Nephelometric principle in compliance with EN ISO 7027	0.05 ... 4000 FNU	(in the range up to 2000 FNU): <1 %	
**ProDSS** Turbidity Sensor	Optical, 90° scatter	0 to 4000 FNU	0 to 999 FNU: 0.3 FNU or ±2% of reading, whichever is greater 1000 to 4000 FNU: ±5% of reading	
**EXO** Turbidity Smart Sensor	Optical, 90° scatter in accordance with ASTM Method D7315-07a	0 to 4000 FNU	0 to 999 FNU: 0.3 FNU or ±2% of reading, whichever is greater; 1000-4000 FNU: ±5% of reading	t_{63} <2 s
**Aqualabo** NTU optical turbidity probe	Diffusion IR at 90°	5 to 4000 NTU	< 5% of the reading	< 1 s
**This work** (1-cm sensor)	Backscattering at 180°	8 – 1000 NTU (extendable to 3000 NTU with a non-linear calibration curve)	0 – 10 NTU: 1 % 10 – 100 NTU: 7% 100 – 1000 NTU: 8%	2 s (variable)
**This work** (1.5-cm sensor)	Backscattering at 180°	1.2 – 500 NTU	0 – 10 NTU: 49% 10 – 100 NTU: 9% 100 – 500 NTU: 7%	2 s (variable)
**This work** (2-cm sensor)	Backscattering at 180°	0.8 – 300 NTU	0 – 10 NTU: 29% 10 – 100 NTU: 7% 100 – 300 NTU: 8%	2 s (variable)

Table S2. Effect of O_2 on the emission lifetimes of the reference and indicator layers of the turbidity sensor.[a,b]

	τ_{air} (ns)	τ_{N_2} (ns)	τ_{O_2} (ns)
BCT/PVC	10.3	10.5	10.5
$[Ru(dpp)_3]^{2+}$/PCA	4807	5382	3870

[a] Uncertainty values are ± 1% for the single exponential decays (PVC) and ± 2% for the biexponential decays (PCA).

[b] Preexponential weighted lifetime ($\tau_M = \Sigma\tau_i B_i/\Sigma B_i$), where B_i is extracted from the best fit of the experimental date to the biexponential decay function ($I_L(t) = A_0 + \Sigma B_i \exp(-t/\tau_i)$).

Additional figures

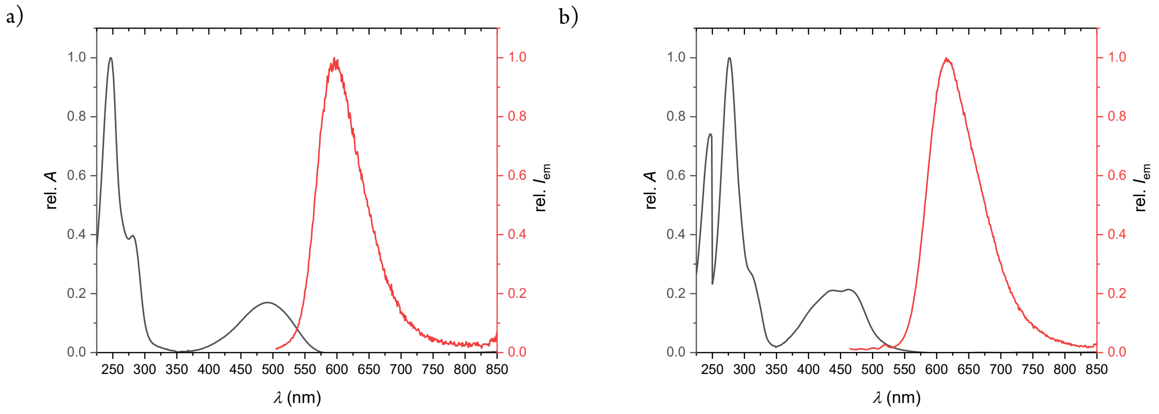

Figure S2. a) Relative absorption and fluorescence of BCT in 1,2-dichloroethane. b) Relative absorption and luminescence of $[Ru(dpp)_3]^{2+}$ in acetonitrile.

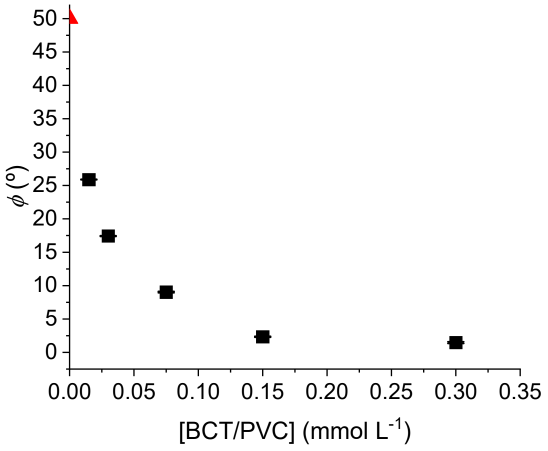

Figure S3. Phase shift values of the turbidity sensor (2-cm sensor head) in clear water using BCT/PVC layers of different concentrations (the latter refers to the dye concentration in the polymer solution before the film formation). The red triangle is the phase shift of $[Ru(dpp)_3]^{2+}$/PCA without perturbation from BCT.

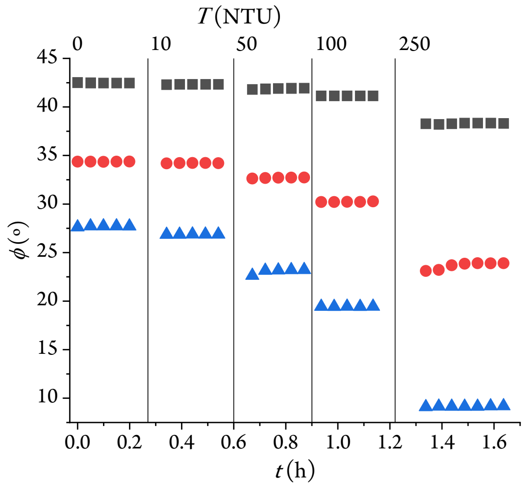

Figure S4. Response of the 1-, 1.5- and 2-cm pathlength turbidity sensor terminals to 0, 10, 50, 100 and 250 NTU formazin suspensions at 25 °C.

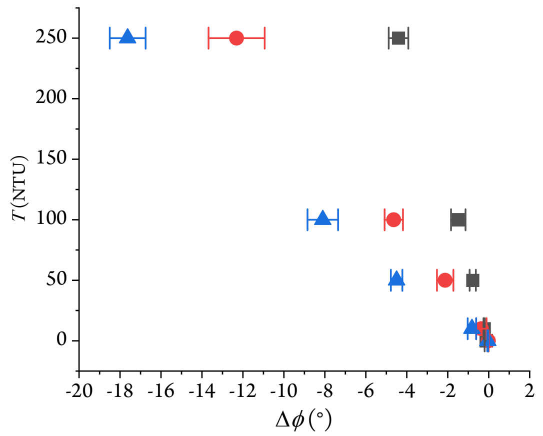

Figure S5. Reproducibility of three measurements of turbidity standards over a 3-month period with regular disassembling and assembling of the detachable bottom of the sensor housing (Figure 1, 1-cm sensor) and with additional removal of the monolith holder (Figure 1, 1,5-cm and 2-cm sensors).

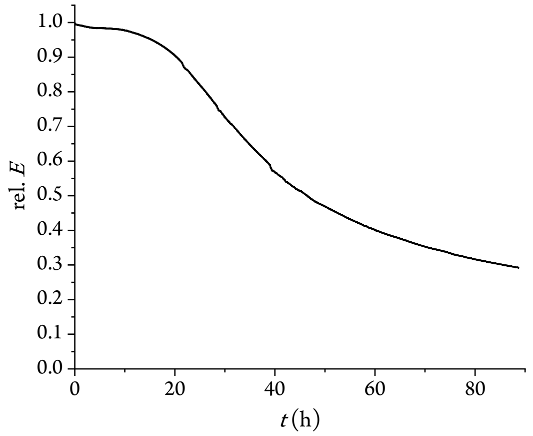

Figure S6. Relative integrated emission intensity of the BCT layer in an accelerated photobleaching test.

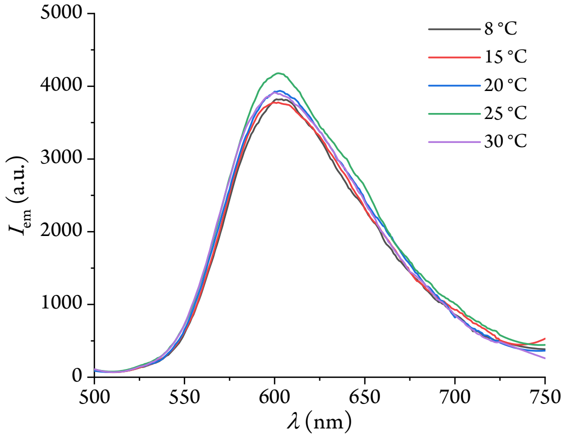

Figure S7. Fluorescence spectrum of BCT in 1,2-dichloroethane at different temperatures.

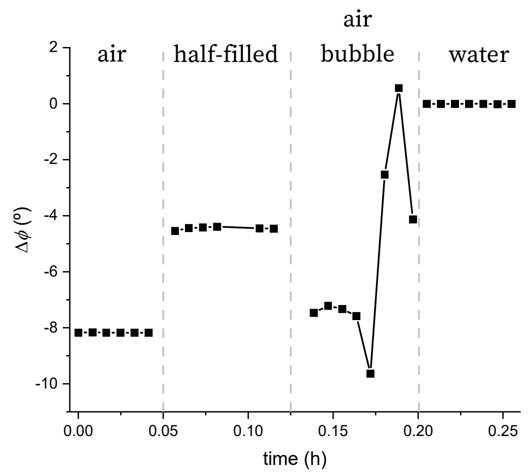

Figure S8. Effect of filling the 2-cm sensor terminal optical pathlength with air on the phase shift excursion at 0 NTU; "air": only air between the glass windows; "half-filled": water filling half of the optical path; "air bubble": bubble crossing the optical path; "water": sensor under normal operation.

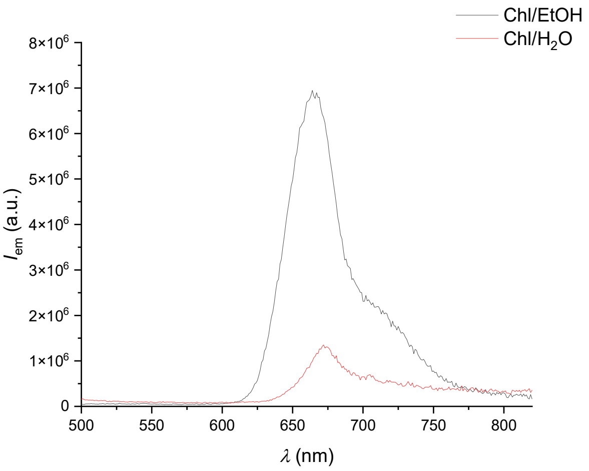

Figure S9. Fluorescence spectrum of a 0.33 μg mL^{-1} chlorophyll solution in absolute EtOH (Chl/EtOH, black) and in 10% EtOH–H_2O (v/v) (Chl/H_2O, red).

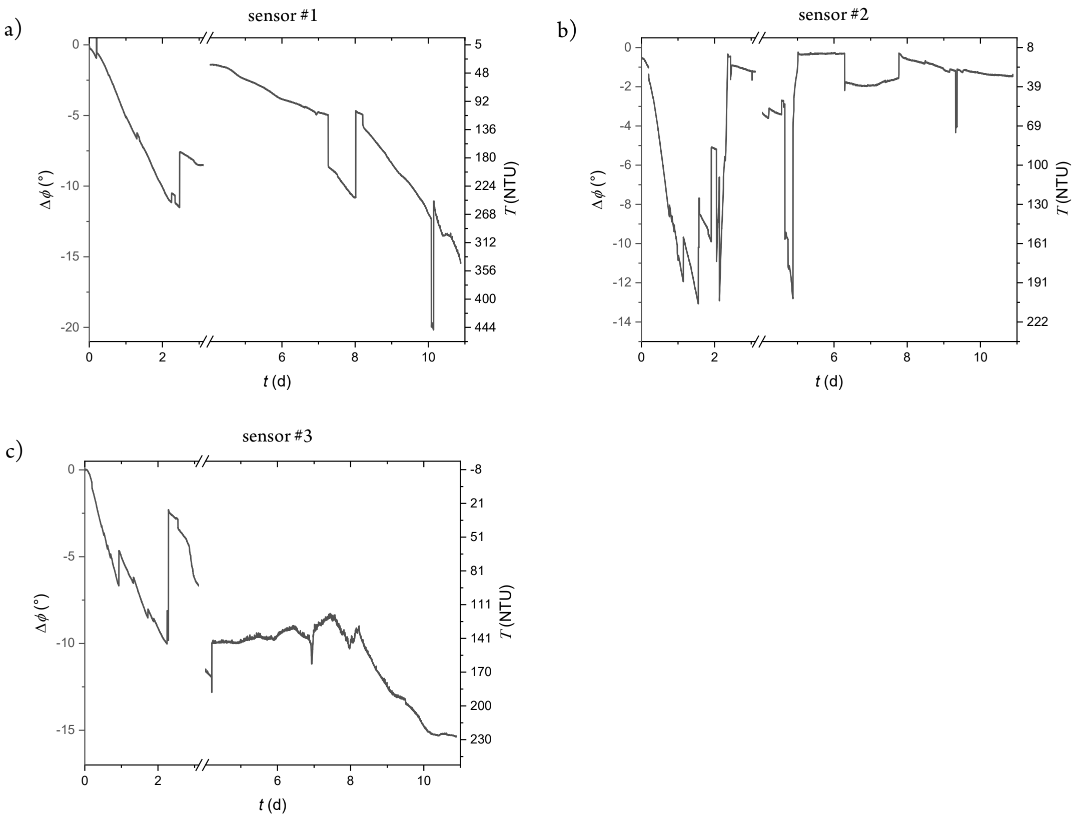

Figure S10. Phase shift excursion and the corresponding turbidity values of the 2-cm sensors #1 (a), #2 (b) and #3 (c) during the 11-day in situ testing at the cul-de-sac pipe.

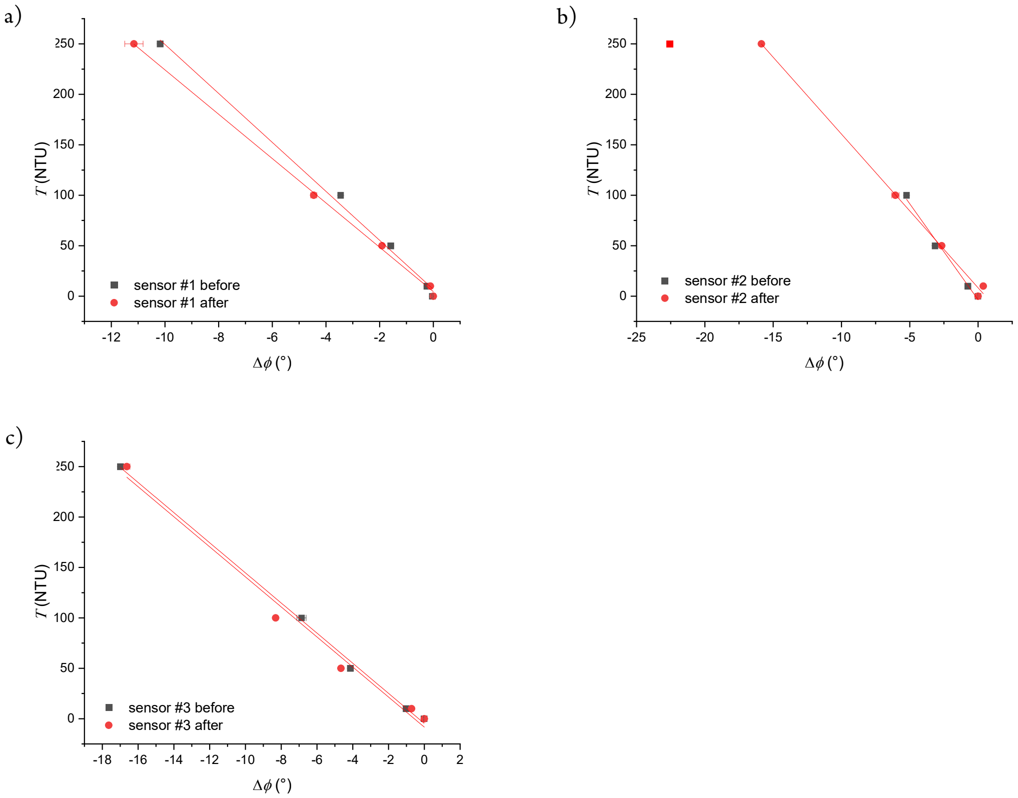

Figure S11. Calibration of the three 2-cm sensors at the start and after the 11-day in situ testing. a) Sensor #1: $T = 7 - 24.3\Delta\phi$ ($R^2 = 0.995$) and $T = 4 - 22.0\Delta\phi$ ($R^2 = 0.999$), before and after the measurement, respectively. b) Sensor #2: $T = -4 - 19.0\Delta\phi$ ($R^2 = 0.989$) and $T = 8 - 15.2\Delta\phi$ ($R^2 = 0.997$), before and after the measurement, respectively ($\Delta\phi$ at 250 NTU was not used for the calibration before the measurement due to an unnoticed air bubble). c) Sensor #3: $T = -5 - 14.9\Delta\phi$ ($R^2 = 0.998$) and $T = -8 - 14.9\Delta\phi$ ($R^2 = 0.985$), before and after the measurement, respectively.

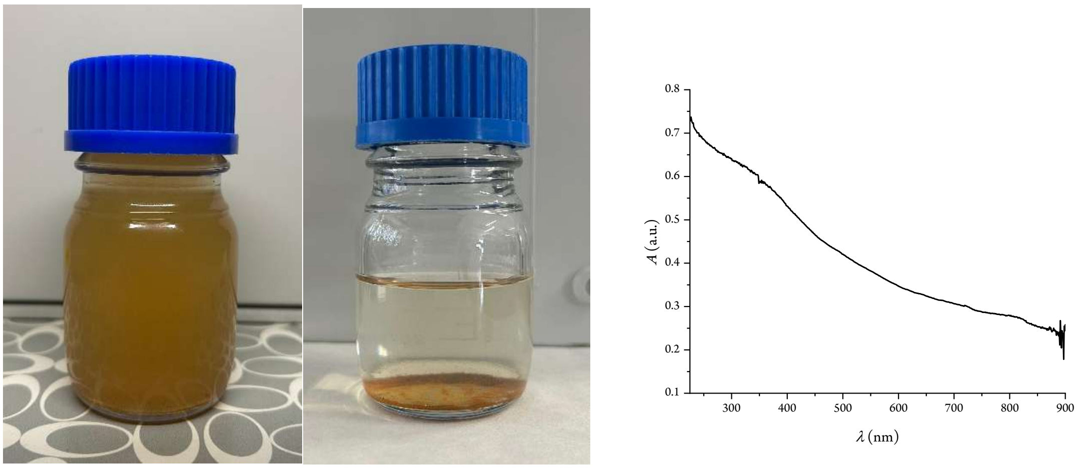

Figure S12. Left: Bottle with suspended particles after fully opening the valve. Middle: Bottle after settling for 2 days. Right: Absorption spectra of the suspended particles.

NMR

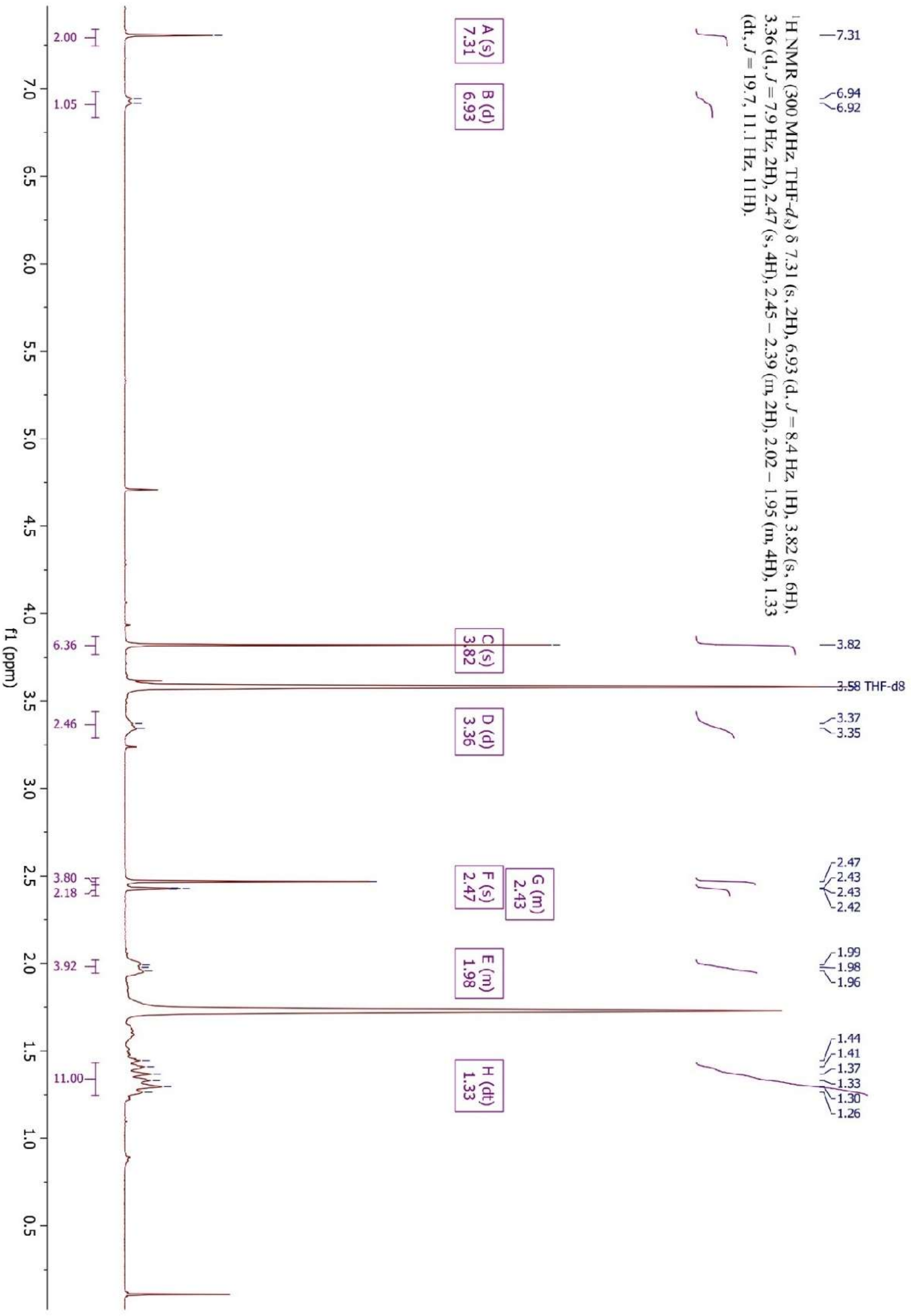

Figure S13. 300-MHz ^{1}H NMR of BCT in THF-d_8.

References

(1) *ISO 7027-1:2016*. ISO. https://www.iso.org/standard/62801.html (accessed 2024-09-26).

(2) Urriza-Arsuaga, I.; Bedoya, M.; Orellana, G. Luminescent Sensor for O_2 Detection in Biomethane Streams. *Sens. Actuators B: Chem.* **2019**, *279*, 458–465. https://doi.org/10.1016/j.snb.2018.09.108.

(3) Kim, J.; An, J. M.; Jung, Y.; Kim, N. H.; Kim, Y.; Kim, D. Red-Emitting SBBF (Single-Benzene-Based Fluorophore)-Silica Hybrid Material: One-Pot Synthesis, Characterization, and Biomedical Applications. *Nanomaterials* **2021**, *11*, 2036. https://doi.org/10.3390/nano11082036.

(4) Lichtenthaler, H. K. Chlorophylls and Carotenoids: Pigments of Photosynthetic Biomembranes. In *Methods in Enzymology*; Plant Cell Membranes; Academic Press, 1987; Vol. 148, pp 350–382. https://doi.org/10.1016/0076-6879(87)48036-1.

(5) Haigh-Flórez, D.; Cano-Raya, C.; Bedoya, M.; Orellana, G. Rugged Fibre-Optic Luminescent Sensor for CO_2 Determination in Microalgae Photoreactors for Biofuel Production. *Sens. Actuators B: Chem.* **2015**, *221*, 978–984. https://doi.org/10.1016/j.snb.2015.07.052.

(6) Bedoya, M.; Díez, M. T.; Moreno-Bondi, M. C.; Orellana, G. Humidity Sensing with a Luminescent Ru(II) Complex and Phase-Sensitive Detection. *Sens. Actuators B: Chem.* **2006**, *113*, 573–581. https://doi.org/10.1016/j.snb.2005.07.006.
